# Micropeptides Encoded by Noncoding RNAs: Biological Functions and Roles in Diseases

**DOI:** 10.34133/research.0967

**Published:** 2025-10-30

**Authors:** Jinghua Kong, Xinwan Su, Cefan Zhou, Weiqiang Lin, Aifu Lin, Jingfeng Tang

**Affiliations:** ^1^ National “111” Center for Cellular Regulation and Molecular Pharmaceutics, Key Laboratory of Fermentation Engineering (Ministry of Education), Hubei University of Technology, Wuhan, China.; ^2^ Department of Respiratory and Critical Care Medicine, Center for RNA Medicine, the Fourth Affiliated Hospital of School of Medicine, and International School of Medicine, International Institutes of Medicine, Zhejiang University, Yiwu, Zhejiang, China.; ^3^ Key Laboratory of Cancer Prevention and Intervention, China National Ministry of Education, Hangzhou, Zhejiang, China.

## Abstract

Traditionally considered noncoding, various classes of noncoding RNAs (ncRNAs)—including long noncoding RNAs (lncRNAs), circular RNAs (circRNAs), primary microRNAs (pri-miRNAs), ribosomal RNAs (rRNAs), and mRNA untranslated regions (UTRs)—have recently been shown to harbor micropeptide-encoding capacity. These functionally versatile micropeptides participate in various cellular processes spanning RNA modification, transcription regulation, splicing machinery, protein translation, and posttranslational modifications. This review systematically examines 3 aspects of ncRNA-derived micropeptides: their genomic origins and biogenesis, mechanistic roles in cellular physiology, and implications in human pathologies including oncogenesis, cardiovascular disorders, and neurodegenerative conditions. We highlight emerging potential as novel therapeutic targets and diagnostic biomarkers. Furthermore, we also discuss current methodologies for micropeptide and functional characterization. In summary, the systematic identification and annotation of disease-related ncRNA-encoded micropeptides has opened up a new milestone in the field for the development of novel targeted therapies and personalized disease treatment strategies.

## Introduction

Micropeptides encoded by small open reading frames (sORFs) have recently been identified as biologically active mediators [1,2]. These sORFs are found in noncoding RNAs (ncRNAs), which constitute approximately 98% of the human transcriptome [3]. Notably, it has been demonstrated that certain ncRNAs, such as long noncoding RNAs (lncRNAs) [4], circular RNAs (circRNAs) [5], primary microRNAs (pri-miRNAs) [6,7], ribosomal RNAs (rRNAs) [8], and untranslated regions (UTRs) of mRNAs [9], demonstrate micropeptide-encoding capacity. These micropeptides, also referred to as microproteins or short open reading frame-encoded peptides (SEPs), are generally less than 100 amino acids (aa) in length and exhibit diverse cellular and physiological functions [10]. Notably, a small number of ncRNAs can be translated into peptides exceeding 100 aa in length, such as circRNAs [11].

Integrated multiomics approaches (e.g., transcriptomics, proteomics, and translatomics) have enabled the identification of ncRNA-derived micropeptides and implicated them in human diseases, including cancer. They regulate critical physiological and pathological functions such as muscle development, tumor progression, metabolism, immune response, and translation network regulation [12]. Rapid advances in computational prediction and experimental techniques have led to the discovery of many previously unknown micropeptides, particularly in the human microbiome, where they may serve as novel drug targets for antibiotics [13].

Emerging studies have shown that circRNAs initiate translation without a 5′ cap structure, producing micropeptides that regulate malignant tumor development and metabolic reprogramming. For instance, circPETH-147aa, encoded by circPETH, enhances glycolysis, metabolic plasticity, migration, invasion, and metastasis in hepatocellular carcinoma (HCC) cells [14]. LncRNA-derived micropeptides play a critical role in various disease processes, such as tumor metabolism, transcriptional regulation, translational and posttranslational regulation, and signal transduction [15,16]. While research on miRNA-encoded peptides (miPEPs) has primarily focused on plants, a small number of miPEPs have been identified in human cells and are closely linked to human diseases [17]. Additionally, MOTS-c, a 16-aa micropeptide encoded by an sORF in the mitochondrial DNA 12*S* rRNA region, improves insulin sensitivity and energy metabolism via the folate–adenosine monophosphate-activated protein kinase (AMPK) pathway, offering a novel target for treating metabolic diseases [8]. The 5′UTR of the yeast transcription factor *GCN4* contains multiple upstream open reading frames (uORFs), whose translation dynamically regulates *GCN4* synthesis [18]. In neuroblastoma (NB), 4,954 translatable uORFs have been identified, and their encoded micropeptides can regulate the translation of the main coding sequence [19]. In summary, ncRNA-encoded micropeptides perform crucial biological functions, and elucidating their roles in diseases will accelerate the identification of clinically relevant therapeutic targets.

This review briefly summarizes the sources of micropeptides derived from ncRNAs, with a particular focus on the biological functions of micropeptides encoded by lncRNAs, circRNAs, pri-miRNAs, and other translatable ncRNAs, as well as their regulatory mechanisms in cancer and other diseases. We also outline cutting-edge methodologies and future research directions for systematic micropeptide identification and mechanistic exploration.

## Identification Methods of NcRNA-Encoded Micropeptides

**Fig. 1.**
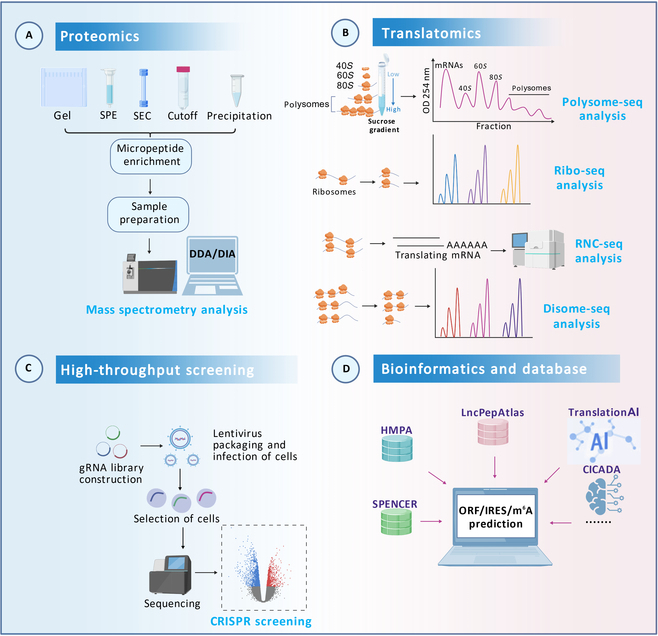
Identification methods for micropeptides. (A) Discovery of micropeptides from proteomics by MS. The MS technical process includes sample pretreatment, micropeptide enrichment, MS detection, data acquisition, and raw data analysis. (B) Micropeptide identification from translatomics by ribosome-associated sequencing technologies. The typical workflow involves capturing ribosome-protected mRNA fragments through enzymatic digestion or centrifugation, followed by fragment purification, library construction, and high-throughput sequencing analysis. (C) High-throughput screening of micropeptides using CRISPR. CRISPR-Cas9 knockout technology for screening functional micropeptides. The main workflow includes gRNA library design, lentivirus packaging and library delivery, phenotypic screening, and sequencing analysis. (D) Prediction of micropeptide-encoding potential using bioinformatics tools and databases.

Technical limitations have historically posed challenges to discovering ncRNA-encoded micropeptides. Early studies often overlooked these micropeptides due to the lack of efficient detection methods. However, with technological advancements, novel methods have emerged for identifying micropeptides [12] (Fig. 1). For example, mass spectrometry (MS)-based proteomics technology can precisely detect the mass of proteins and peptides, aiding in micropeptide identification. Additionally, ribosome-based translatomics technology, which analyzes ribosome positions on mRNA, reveals dynamic translation changes and provides crucial clues for micropeptide discovery [20]. Furthermore, high-throughput CRISPR screening enables the systematic identification of key ncRNA-encoded micropeptides and the study of their functions in cell proliferation, metabolic regulation, and disease development [21]. Bioinformatics prediction methods also play a key role by analyzing RNA sequences and structural features to predict potential micropeptide-encoding regions [22]. These identification methods have accelerated the discovery of ncRNA-encoded micropeptides, providing a foundation for exploring their biological functions and discovering new therapeutic targets. The advantages, disadvantages, costs, and application scope of these micropeptide identification technologies are listed in Table 1.

**Table 1.** Comparison of micropeptide identification technologies

Technologies	Strengths	Challenges	Cost	Range of application
Mass spectrometry- based proteomics technology	Support confirmation of actual expressed micro-peptide sequences and accuracy quantification of their abundance	Challenging to detect low- expression micro- peptides	High	Validate translation of predicted micro- peptides and quantify expression
Ribosome-based translatomics technology	Provides precise and comprehensive ribosomal positioning information and detailed analysis of translational regulatory events, such as the translation of sORFs	Require complex computational pipelines for analysis	High	Identify sORFs and novel micropeptide
High-throughput screening based on CRISPR	Investigate thousands of targets in a single experiment and directly link sORF to a phenotypic outcome	Without direct evidence of micro- peptide translation	Medium-high	Identify micro- peptides essential for specific pathways
Bioinformatics-based prediction	Low cost and rapidly scan numerous genomes to generate micropeptide candidate gene lists	Predictions are only as good as the underlying algorithms and training data	Low	Initial genome or transcriptome-wide scans for putative sORFs

sORFs, small open reading frames

### Bioinformatics-based prediction

Bioinformatics-based prediction of ncRNA-encoded micropeptides has rapidly developed as an essential tool for revealing their translation potential (Fig. 1D). ORF prediction algorithms combined with Ribo-seq data can efficiently identify hidden sORFs in ncRNAs and assess their translation potential [23]. Moreover, for studying nonclassical translation mechanisms, internal ribosome entry site (IRES) prediction tools locate non-AUG-initiated translational regions, revealing the translation capacity of ncRNAs [24]. Furthermore, N6-methyladenosine (m^6^A) modification identification has emerged as a key direction; predicting m^6^A modification sites, combined with experimental confirmation, demonstrates their role in promoting nonclassical translation by recruiting initiation factors [25]. As understanding deepens, researchers have integrated multi-omics data from comprehensive databases to construct global maps of micropeptides encoded by different ncRNAs.

#### ORF prediction

ORFs are DNA segments encoding a protein, starting at a start codon (e.g., ATG/AUG) and ending at a stop codon. sORFs typically refer to ORFs shorter than 100 codons in eukaryotes and shorter than 50 or 70 codons in prokaryotes. Due to limitations in genetic prediction, sORFs are regarded as the genome’s “dark matter” [26]. Besides DNA fragments, sORFs can originate from various RNA types. Many sORFs originate from ncRNAs, such as lncRNAs [27]. The sORFs within these ncRNAs may have acquired coding functions through evolution, enabling them to translate functional micropeptides [28]. Rapid technological advances have created multiple tools for predicting and validating the translation potential of ncRNAs with sORFs.

Tools like CircRNADb [29], CircBank [30], and CircCode [31] annotate ORFs and IRES information for circRNAs with coding potential. Prediction tools, including Phylogenetic Codon Substitution Frequencies (PhyloCSF), csORF-finder, and GWIPS-viz, also predict sORFs with coding potential [32–42] (Table 2).

**Table 2.** Bioinformatic tools for ORFs

Name	Main functions	Category	Species	Website	Ref.
PhyloCSF	Distinguish protein-coding and ncRNAs, and ORF prediction	Transcriptomic profiling	Multi-species	http://compbio.mit.edu/PhyloCSF	[32]
csORF-finder	Specialize for sORF prediction	Transcriptomic profiling	Multi-species	https://github.com/xiaofengsong/csORF-finder	[33]
GWIPS-viz	Identification of uORF/sORF by comparison with RNA sequencing	Transcriptomic profiling	Multi-species	https://gwips.ucc.ie	[34]
CircRNADb	Specialize for collecting human exonic circRNA and annotates translation potential	circRNA	*Homo sapiens*	http://reprod.njmu.edu.cn/circrnadb	[29]
CircBank	Integrate 140,790 human circRNAs, predicted ORFs by CPAT, and predicted IRES elements using IRESfinder	circRNA	*Homo sapiens*	https://www.circbank.cn	[30]
CircCode	Specialize for circRNA-encoded micropeptide prediction through ORF/IRES identification	circRNA	Multi-species	https://github.com/PSSUN/CircCode	[31]
smORFer	Specialize for sORF detection with modular	Genomic profiling	Multi-species	https://github.com/AlexanderBartholomaeus/smORFer	[36]
uPEPperoni	Specialize for identifying uORFs and uORF-encoded peptides	Transcriptomic profiling	Multi-species	http://upep-scmb.biosci.uq.edu.au	[37]
RiboTaper	Specialize for identifying translated ORFs	Transcriptomic profiling	Multi-species	https://github.com/ohlerlab/RiboTaper	[38]
Ribotricer	Support for identifying novel ORFs	Transcriptomic profiling	Multi-species	https://github.com/smithlabcode/ribotricer	[39]
SPECtre	Quantify identification of translational active ORFs	Transcriptomic profiling	Multi-species	https://github.com/mills-lab/spectre	[40]
RiboHMM	Support for identifying uORFs/sORFs	Transcriptomic profiling	Multi-species	https://github.com/rajanil/riboHMM	[41]
ORFquant	Quantify translation of ORFs within transcript space	Transcriptomic profiling	Multi-species	https://github.com/lcalviell/ORFquant/releases/tag/1.02	[42]

ncRNAs, noncoding RNAs; ORFs, open reading frames; sORFs, small open reading frames; uORFs, upstream open reading frames; CPAT, Coding Potential Assessment Tool; IRES, internal ribosome entry site

#### IRES prediction

IRES is a specialized RNA sequence that mediates ribosome-initiated translation internally within lncRNAs or circRNAs, independently of the 5′ cap structure [43]. It can facilitate circRNA translation under specific conditions to generate novel functional proteins [44]. Multiple tools, such as IRESite, IRESbase, and IRESfinder prediction databases, predict IRES sequences to determine the mechanism of translation of ncRNAs [45–47]. Besides, IRESPred is one of the earliest machine learning IRES prediction tools, but it is only applicable to linear RNA and has limitations in negative sample construction [48]. IRESite, IRESPred, and IRESfinder are the most frequently used IRES prediction tools. IRESpy is an IRES prediction tool developed based on the XGBoost machine learning model. IRESpy is currently one of the most accurate, fastest, and most user-friendly IRES prediction tools available for high-throughput screening of potential IRES in human 5′UTRs. It has the disadvantage of being applicable only to linear RNA [49]. Moreover, DeepCIP was developed specifically to predict circRNA IRES based on multimodal deep learning [50].

#### m^6^A modification prediction

In addition to IRES-dependent translation, m^6^A modification-driven translation represents another cap-independent mechanism for ncRNA translation [51]. The m^6^A modification of nucleotides regulates critical biological processes such as RNA splicing, translation, and degradation, and is closely associated with various human diseases, including cancer. Accumulating evidence shows that m^6^A modification of ncRNAs can initiate sORF translation [52]. However, m^6^A modifications of other ncRNAs, including rRNAs and the UTRs of mRNAs, have not been reported to initiate sORF translation. To systematically study the m^6^A modification of RNA, researchers have developed tools to predict the m^6^A modification sites. For example, DeepM6Aseq, a neural network model based on m^6^A individual-nucleotide-resolution cross-linking and immunoprecipitation sequencing (miCLIP-seq) data, predicts single-base m^6^A modifications, captures surrounding biology, and visualizes m^6^A sites [53]. Furthermore, the SRAMP tool supports ncRNA sequence analysis by identifying m^6^A sites in RRACH sequences using machine learning algorithms [54]. Pierattini et al. [55] recently identified the m^6^A methylation site of the lncRNA SINEUP using SRAMP and demonstrated that m^6^A modification is required for SINEUP activity. Additionally, iRNA-m6A [56], and M6APred-EL [57] are also key tools for predicting m^6^A modification sites (Table 3).

**Table 3.** Bioinformatic tools for IRES and m^6^A modification

Name	Main functions	Category	Species	Website	Ref.
IRESite	Provide detailed information on the IRES regions of eukaryotic viruses and cells	Linear RNA or circRNA	Multi-species	http://www.iresite.org	[45]
IRESbase	Include 1,328 IRESs and focus on IRES information in circRNA and lncRNA	circRNA or lncRNA	Multi-species	http://reprod.njmu.edu.cn/cgi-bin/iresbase/index.php	[46]
IRESfinder	Suitable for circRNA IRES site prediction	Linear RNA or circRNA	Multi-species	https://github.com/xiaofengsong/IRESfinder	[47]
IRESPred	Early IRES prediction tool based on SVM	Linear RNA	Multi-species	http://bioinfo.net.in/IRESPred/	[48]
IRESpy	High accuracy and fast prediction IRES using XGBoost machine learning	5′UTR	Multi-species	https://irespy.shinyapps.io/IRESpy/	[49]
DeepCIP	Specialize for circRNA IRES prediction by deep learning	circRNA	*Homo sapiens*	https://github.org/zjupgx/DeepCIP	[50]
DeepM6Aseq	Suitable for high- throughput m^6^A site prediction	Epitranscriptomics profiling	Multi-species	https://github.com/chenkenbio/DeepM6ASeq	[53]
SRAMP	Specialize for m^6^A modification sites online prediction	mRNA or lncRNA	Multi-species	http://www.cuilab.cn/sramp	[54]
iRNA-m6A	Provide m^6^A modification sites prediction	Epitranscriptomics profiling	Multi-species	http://lin-group.cn/server/iRNA-m6A/	[56]
M6APred-EL	High accuracy prediction m^6^A sites using ensemble learning	Epitranscriptomics profiling	Multi-species	https://github.com/chr2117216003/M6APred-EL	[57]

SVM, support vector machines; XGBoost, eXtreme gradient boosting; m^6^A, N6-methyladenosine

### Computational biology, database, and software

Various computational algorithms, databases, and software are currently used to study ncRNA-encoded micropeptides. In eukaryotic cells, RNA translation initiation and termination are highly regulated biological processes directly linked to abnormal protein expression and function. However, existing gene annotation and experimental techniques still face major challenges in identifying translation sites and predicting potential coding regions, especially for sORFs in ncRNAs like lncRNAs, which traditional methods often fail to capture. Fan et al. [58] constructed a deep neural network-based TranslationAI model to accurately predict the RNA translation initiation site (TIS) and translation termination site (TTS). They used TranslationAI to systematically predict potential new ORFs in the human transcriptome, including 673 upstream ORFs (uORFs), 127 downstream ORFs (dORFs), and 3,794 new TIS–TTS pairs in lncRNAs. Multiple newly predicted ORFs are supported by proteomic data and functional studies, suggesting that ncRNAs may have extensive undiscovered coding potential. To comprehensively annotate lncRNA upstream regulation and integrate translational evidence for lncRNA-encoded micropeptides across species, Zhou et al. [59] created the LncPepAtlas platform. This platform analyzes a dataset of 2,655 ribosomal analyses from 9 species, providing evidence for the translation of numerous lncRNA-encoded micropeptides. Furthermore, the LncPep and TransLnc databases integrate experimental and computational prediction data, offering powerful support for studying lncRNA translational potential [60,61].

Researchers leverage various algorithmic strategies to predict the coding potential of different ncRNA types. Fan et al. [62] innovatively proposed CICADA, a circRNA coding capacity and product detection algorithm, which utilizes machine learning to identify potentially translatable circRNAs. CICADA also offers a proprietary strategy for identifying circRNA-encoded products, serving as a peptide library for MS searches. Researchers discovered 222 circRNAs with coding potential in esophageal squamous cell carcinoma (ESCC) using CICADA and identified translation products for 4 circRNAs via MS.

Li et al. [63] constructed the valuable small protein database SmProt, providing comprehensive characterization of small protein-coding genes across species. Based on 419 Ribo-seq datasets, SmProt screened 638,958 unique small proteins, including many encoded by UTRs and ncRNAs, by cross-integrating information and removing duplicates. It offers a new reference for ncRNA, functional genomics, and clinical research. Luo et al. [64] established SPENCER, an ncRNA-encoded micropeptide database utilizing MS data from cancer patients. SPENCER collected MS data from patient tissues of 15 common cancers, reanalyzing and sorting them to obtain 29,526 cancer-related ncRNA-encoded micropeptides. Besides basic sequence, transcript, gene, and RNA structure annotations, SPENCER provides differential expression and immunogenicity analysis of these micropeptides in tumor versus normal tissues. Although various databases contain sequence and functional information for some ncRNA-encoded micropeptides, the expression and latent functional roles of cancer-related ones require further exploration and integration. Therefore, researchers have recently constructed HMPA, a nonclassical peptide database, to systematically collect and analyze cancer-related nonclassical peptides using large cohorts of publicly available proteomics, transcriptomics, and clinical data. Researchers collected extensive genomic, transcriptomic, and proteomic data, along with noncanonical peptide expression profiles and cancer-related clinical phenotyping data from multiple sources, preprocessed for compatibility and quality. Researchers utilized the Ribotricer tool to construct a reference database containing approximately 10 million sORFs using the Ensembl human genome, providing a basis for subsequent micropeptide discovery. The HMPA database is founded on a robust framework designed to create a reliable reference library, utilizing cancer-related multi-omics data to outline an extensive repository of human cancer-relevant micropeptides. Tens of millions of micropeptides are comprehensively annotated, including basic information, advanced structure prediction, potential peptide interaction networks for biological function, and predictions of expression patterns and clinical prognostic indicators across different cancers [65].

PointNovo is a de novo sequencing software designed for high-resolution MS. This computational model can be utilized in MS analysis at nearly any resolution, facilitating ncRNA-encoded micropeptide discovery. Wang et al. [66] performed de novo peptide sequencing using PointNovo, identifying 1,682 peptides and discovering 2,544 human sORFs. Recently, new software tools like DeepSearch [67] and DiNovo [68] have been developed to improve peptide identification accuracy, though not yet applied to ncRNA-encoded micropeptides. These innovative software tools provide new avenues for micropeptide discovery. These computer algorithms, databases, and software are listed in Table 4.

**Table 4.** Computational biology, database, and software for micropeptides

Name	Main functions	Category	Species	Website	Ref.
TranslationAI	lncRNA translation potential prediction using deep learning	lncRNA	Multi-species	https://www.biosino.org/TranslationAI/	[58]
LncPepAtlas	Experimentally verified lncRNA- derived micropeptide database	lncRNA	Multi-species	http://www.cnitbiotool.net/LncPepAtlas/	[59]
LncPep	lncRNA coding ability prediction through multi-feature fusion	lncRNA	Multi-species	http://www.shenglilabs.com/LncPep/	[61]
TransLnc	Comprehensive for lncRNA translation potential prediction	lncRNA	Multi-species	http://bio-bigdata.hrbmu.edu.cn/TransLnc/	[60]
CICADA	Assess circRNA the protein-coding potential	circRNA	Multi-species	https://github.com/SunLab-biotool/CICADA	[62]
HMPA	Provide annotations, predict subcellular localization, physicoche mical properties, representative MS spectra, structures and mPPI interaction in 8 types of cancer	Multi-omics profiling	*Homo sapiens*	http://hmpa.zju.edu.cn	[65]
SmProt	Integrate small protein information from multiple sources, provide basic annotations including species origin, tissue/cell line origin, data source, etc.	Multi-omics profiling	Multi-species	http://bigdata.ibp.ac.cn/SmProt	[63]
sORFs.org	Specialize for identifying sORFs using Ribo-seq and store the results in a public database	Transcriptomic profiling	Multi-species	https://www.sorfs.org	[82,83]
OpenProt	Analyze Ribo-seq and MS-based proteomics data to collect evidence for predicted protein expression at the translational and protein levels	Multi-omics profiling	Multi-species	https://www.openprot.org/	[84]
SPENCER	Analyze the regulatory role of micropeptides through expression profiles, interaction, and functional annotation in cancer	Proteomics profiling	*Homo sapiens*	http://www.spencer.renlab.org	[64]

MS, mass spectrometry; mPPI, micropeptide–protein network; Ribo-seq, ribosome sequencing

### MS-based proteomics technology

MS analysis enables direct detection of micropeptides translated from sORFs, providing direct evidence for ncRNA encoding. Although MS is the gold standard for identifying new peptides or proteins, detecting micropeptides remains challenging due to their small molecular weight, low abundance, and poor annotation [10,69]. The typical steps for MS identification of micropeptides include enrichment, sample preparation, data collection, and raw data analysis [70].

Several optimization and enrichment methods improve the accuracy of MS assays for sORF-translated micropeptides. The protein structure denaturation by boiling eliminates peptidase and protease activity, preventing intracellular micropeptide degradation. Micropeptide enrichment can be performed using various methods depending on size, hydrophobicity, and charge properties, such as sodium dodecyl sulfate–polyacrylamide gel electrophoresis (SDS-PAGE) gel separation [71], molecular weight cutoff filters [72,73], organic solvent precipitation, C8 solid-phase extraction (SPE) column [72,74], and size exclusion chromatography (SEC) [70,75].

In addition to improved enrichment methods, combining MS with other techniques also improves micropeptide detection accuracy. Previous studies identified 90 SEPs in human K562 cells, of which 86 were newly discovered peptides using liquid chromatography–tandem MS (LC-MS/MS) combined with RNA sequencing [76]. Further studies show that ultrafiltration efficiently enriches novel peptide signals while markedly reducing interference from large molecular proteins [75]. Recent studies have also demonstrated that ultrafiltration technology coupled with mass spectrometry can identify a large number of micropeptides. Using 30-, 10-, and 3-kDa ultrafiltration tubes to separate proteins, followed by LC separation and MS analysis, researchers identified 8,945 novel peptides in gastric cancer (GC) tissues, AGS GC cell lines, and normal gastric tissues [22]. A recent study identified numerous micropeptides in clinical HCC samples based on the ribosome sequencing (Ribo-seq) database and tandem MS [77]. Improving micropeptide enrichment methods or combining MS with other techniques is crucial for characterizing micropeptide presence (Fig. 1A).

Before MS analysis, samples are typically subjected to enzymatic digestion, breaking down proteins into smaller peptides, usually using trypsin or other proteases such as LysargiNase, chymotrypsin, Asp-N, Lys-C, or Glu-C [70,78]. The use of multiple enzymes in combination facilitates the discovery of micropeptides. For example, Kaulich et al. [78] identified only 55 small proteins and 22 SEPs with trypsin alone, but 63 small proteins and 28 SEPs using multiple enzymes. However, since micropeptides encoded by sORFs typically have low molecular weights, whether to digest them enzymatically depends on the research objective. To ensure the completeness of the peptide profile, Ma et al. [73] used undigested protein samples to identify an additional 195 micropeptides in K562 cells. They applied this method to other cell lines and human tissues, identifying 237 new micropeptides. Similarly, Wang et al. [66] identified 241 human micropeptides from 2,544 human sORFs using undigested samples.

Data-dependent acquisition (DDA)-based MS data acquisition modes have identified several micropeptides [74]. The data-independent acquisition (DIA) data acquisition mode is employed to identify low-abundance molecules alongside high-abundance ones, addressing the limitations of DDA in detecting low-abundance molecules [79]. Tzani et al. [80] identified 40 micropeptides in Chinese hamster ovary (CHO) cells using DIA combined with machine learning. Recently, Van der Spek et al. [81] created a directDIA workflow addressing DIA data complexity. Several databases based on MS raw data aid micropeptide identification, including SmProt [63], sORFs.org [82,83], OpenProt [84], and SPENCER [64].

### Ribosome-based translatomics technology

High-throughput sequencing technologies have been developed to analyze and predict the translation of ncRNAs into micropeptides. Translatomics captures and sequences ribosome-associated mRNAs to quantify gene translation across different samples [85]. Ribosome-associated sequencing technologies isolate ribosome–RNA complexes and utilize deep sequencing to obtain information on translated RNA [86] (Fig. 1B).

Ribosome profiling, also known as Ribo-seq [87], studies the translation process by analyzing ribosome distribution on mRNA [88]. It combines ribosome-protected mRNA fragments with high-throughput sequencing to provide detailed information on translation initiation, elongation, and termination. Ribo-seq can identify coding regions genome-wide, filling gaps in gene annotation databases. Previous reports used Ribo-seq combined with cardiac tissue proteomics data to systematically analyze translated ORFs and corresponding proteins/peptides in left ventricular myocardial tissue, identifying 40 circRNAs with translatable potential; further MS data analysis detected micropeptides encoded by 6 of these circRNAs [89]. Emerging research combining Ribo-seq, TIS analysis, and TTS analysis mapped the translatome of *Campylobacter jejuni*, validated the translation of 47 of 55 annotated sORFs, identified 42 new high-confidence sORFs, and further identified the highly conserved small protein CioY [90]. Moreover, ribosome-nascent chain complex sequencing (RNC-seq) analyzes mRNA in translation by centrifuging mRNA bound to ribosomes and performing full-length sequencing [91]. RNC-seq has greater sequencing depth and is more likely to detect circRNA translating micropeptides than Ribo-seq. Zhong et al. [92] utilized RNC-seq coupled with RNA-binding protein immunoprecipitation (RIP) analysis and sequencing, identifying 5 candidate translatable circRNAs: circMET, circSPECC1, circXYLT1, circCDYL, and circRBM33. Furthermore, polysome-sequencing (Polysome-seq) is the gold standard for identifying translational activity levels [93]. Aspden et al. [94] systematically analyzed sORF translation in human, mouse, zebrafish, nematode, and yeast using Polysome-seq. Recently, to address the inability to detect ribosome collisions, researchers developed disome-sequencing (Disome-seq). By enriching ribosomes paused or collided during translation, this technology analyzes double ribosome or collision events to study specific translational regulatory mechanisms [95].

Collectively, these Ribo-seq technologies are essential for ORF studies and identifying potentially translatable ncRNAs.

### High-throughput screening based on CRISPR

As an emerging and vital gene editing technology, CRISPR-Cas9 screening has been applied to systematically characterize functional ncRNA-encoded micropeptides (Fig. 1C). CRISPR/Cas9-mediated knockout screens systematically assessed the functional impact of thousands of micropeptides or proteins in various human cell lines. Chen et al. [96] identified 703 micropeptides encoded by sORFs or uORFs in human induced pluripotent stem cells (iPSCs) and K562 cells using CRISPR-Cas9 knockout. Similarly, Hofman et al. [9] utilized CRISPR-Cas9 to screen 323 microproteins associated with medulloblastoma cell survival across 7 cell lines. They found that MYC family oncogenes may mediate the up-regulation of the micropeptide ASNSD1-uORF, which interacts with the prefoldin-like chaperone complex, promoting medulloblastoma cell survival. Schlesinger et al. [97] identified 83 micropeptides from 11,776 sORFs critical for A375 cancer cell line growth using high-throughput CRISPR/Cas9 knockout screening. A genome-wide CRISPR inactivation screen identified the muscle-specific micropeptide Myomixer, localized to the cell membrane, which promotes myogenic cell fusion and interacts with another fusogenic membrane protein, Myomaker [98].

It has been demonstrated that cell proliferation-based CRISPR screening identified 1,161 ncRNA-derived micropeptides that are closely associated with GC cell proliferation, and verified the existence and function of these micropeptides using Flag tag knock-in and antibody preparation. Furthermore, using a newly developed artificial intelligence (AI)-based structural prediction-driven peptide–protein interaction network analysis framework, researchers confirmed that the micropeptides pep1-nc-OLMALINC, pep5-nc-TRHDE-AS1, pep-nc-ZNF436-AS1, and pep2-nc-AC027045.3 influence tumor cell growth and survival by regulating mitochondrial complex assembly, cholesterol metabolism, and other pathways [22]. Recently, Nah et al. [99] used CRISPR-Cas9 screening to identify SMIM26, encoded by lncRNA LINC00493, which regulates protein translation and oxidative phosphorylation in K562 cells.

In summary, the emergence of novel biotechnological tools and computational AI techniques has made the discovery and functional characterization of ncRNA-encoded micropeptides more comprehensive and effective. Combining these technologies not only advances ncRNA-encoded micropeptide research but also provides new targets and strategies for disease diagnosis and treatment.

## Types of Translatable NcRNA-Encoded Micropeptides

Over the years, many different types and sizes of ncRNAs have been consistently identified based on high-throughput RNA sequencing and other sequencing technologies. Although initially thought to lack translational potential, accumulating studies over the past decade have found that some ncRNAs are translated into small regulatory peptides (sPEPs) or micropeptides. These translatable ncRNAs include miRNAs, lncRNAs, and circRNAs. Additionally, a small number of rRNAs and mRNA UTRs also appear to have micropeptide-coding potential, although their functional roles remain largely incompletely understood [100].

### Pri-miRNA-encoded micropeptides

Pri-miRNAs are miRNA precursors. Generally, RNA polymerase II transcribes miRNAs first into pri-miRNAs with a cap structure and polyadenylation [101]. The pri-miRNA hairpin structure is further processed into mature miRNAs. Translatable pri-miRNAs containing sORFs encode functional micropeptides that regulate cell fate. Typically, peptides encoded by pri-miRNAs are referred to as miPEPs. Lauressergues et al. [102] identified for the first time 2 miPEPs in plants, namely, miPEP171b and miPEP165a. Similarly, some miPEPs have been identified in human cells. For example, pri-miRNA-31 encodes miPEP31, which suppresses autoimmunity by promoting regulatory T cell (Treg) differentiation [103]. Moreover, pri-miR-34 encoding miPEP133 was identified in nasopharyngeal carcinoma. The miPEP133 expression was down-regulated in tumors, induced apoptosis, inhibited cancer cell migration and invasion, and suppressed tumor growth in vivo [7,104]. Nevertheless, accumulating evidence suggests that translatable pri-miRNAs encode micropeptides playing significant roles in cell fate regulation, immunosuppression, and tumorigenesis.

### LncRNA-encoded micropeptides

LncRNAs are RNAs with transcripts longer than 200 nucleotides, and early studies suggested that they lack protein-coding ability. Therefore, lncRNAs were initially considered transcription byproducts [105]. However, recent studies reveal that lncRNAs regulate a wide range of physiological and pathological processes in normal tissues and cancer through interactions with DNA, RNA, or protein [106]. For a long time, researchers overlooked the translational potential of sORFs within lncRNAs. Recently, accumulated studies demonstrate that lncRNA sORFs can encode micropeptides [76,107]. Current understanding considers that sORFs, regulated by start and stop codons, undergo translation similarly to mRNAs [108].

A study identified a 34-aa micropeptide, DWORF, in muscle-specific lncRNA transcripts, the third-smallest known full-length protein in the mouse genome. Knockout of *Dworf* in mice affects skeletal muscle contractile function [109]. A previous study found that lncRNA phenotype switching regulator (PSR) was specifically expressed in vascular smooth muscle cells (VSMCs) and markedly up-regulated during vascular remodeling. Additionally, lncPSR encodes the 117-aa micropeptide Arteridin, and its deletion substantially attenuates vascular remodeling induced by carotid artery injury or angiotensin II (Ang II) [110]. Recent studies confirmed that in ESCC, the peptide encoded by KDM4A-AS1 reduces ESCC cell activity and migration capacity and is associated with fatty acid metabolism and redox processes [111]. Previous studies found that the 53-aa micropeptide encoded by the lncRNA HOXB-AS3 inhibits colorectal cancer (CRC) growth, whereas the lncRNA itself does not [112]. Recent studies reported that the micropeptide TPM3P9, encoded by lncRNA TPM3P9, has oncogenic activity in clear cell renal cell carcinoma (ccRCC). The TPM3P9 promotes cell proliferation and tumor growth by enhancing oncogenic RNA splicing [113]. In summary, numerous micropeptides encoded by translatable lncRNAs have been identified and are involved in cancer and other disease processes.

### CircRNA-encoded micropeptides

Although most circRNAs lack coding ability, they can be translated into functional micropeptides under specific conditions [114]. CircRNA translational capacity arises from their binding to polyribosomes and the presence of sORFs, IRES elements, and nucleotides with m^6^A modifications. These features are essential for translation processes independent of classical start codons [115]. Recent studies also indicate that an m^6^A-modified start codon may act as an IRES and influence circRNA translation [116]. According to circRNADb data, 32,914 circRNAs are predicted to encode proteins; up to 16,328 encode more than 100 aa, and up to 7,170 contain IRES elements, indicating that short peptide encoding ability exists in many circRNAs.

Accumulating studies show that circRNAs are translated into functional micropeptides, playing critical roles in cellular physiology [117]. For example, circ-SHPRH encodes SHPRH-146aa, which inhibits malignant cell proliferation in glioblastoma (GBM) [118]. Moreover, circFBXW7 was found to encode FBXW7-185aa, associated with cell cycle regulation [119]. Early studies found that circ-ZNF609 can be translated into proteins promoting myofibroblast proliferation [114]. Another recent study found that circ-ZNF609 encodes ZNF609-250aa, a 250-aa novel protein that exacerbates acute kidney injury (AKI) [120,121] by inhibiting autophagic flow and inducing apoptosis via an AKT/mammalian target of rapamycin (mTOR)-dependent mechanism [122]. Mounting evidence indicates that translatable circRNAs encode micropeptides, expected to become important components of the “dark proteome” and provide new targets for cancer and other diseases.

### Other types of ncRNA-encoded micropeptides

In eukaryotic cells, rRNAs constitute about 80% of total RNA [123]. Eukaryotic ribosomes comprise approximately 80 ribosomal proteins and 4 key rRNAs, which together maintain ribosome structure and are responsible for protein synthesis [124,125]. It is commonly accepted that rRNAs provide a structural scaffold for ribosomal protein localization, ensuring ribosome assembly and function [126]. The process of protein translation exists in all cellular organisms, with rRNAs catalyzing polypeptide synthesis with transfer RNA (tRNA) assistance [127]. It has been shown that sORFs are also present in rRNAs and possess coding capacity [8]. However, attention to the ability of translatable rRNAs to encode micropeptides remains very limited. Consequently, few rRNA-encoded micropeptides have been reported. Mitochondrial derived peptides (MDPs) are a class of mitochondrial micropeptides encoded by rRNAs that function to regulate cellular metabolism. Currently, 8 known MDPs, including Humanin (HN), MOTS-c, and small humanin-like peptides 1 to 6 (SHLPs 1 to 6), can mitigate disease progression, including cardiovascular disease (CVD), Alzheimer’s disease (AD), diabetes, and macular degeneration [128].

In eukaryotes, mature mRNA has 3 parts: the 5′UTR, the coding region coding sequence (CDS), and the 3′UTR [129–131]. The 5′UTR at the mRNA’s 5′ end is critical for stability and translation efficiency. The CDS contains triplet codons corresponding to amino acids and serves as the blueprint for protein synthesis. The 3′UTR at the mRNA’s 3′ end is also involved in stability and translational regulation [132]. The 5′UTR and 3′UTR play significant roles in the posttranscriptional regulation of eukaryotic gene expression [133]. Although translation is usually initiated from the ATG start codon of CDS, it may also be initiated at the 5′UTR or downstream of the termination codon in the 3′UTR under certain circumstances [134]. These ORFs, located upstream or downstream of the CDS, are approximately 100 nucleotides in length and are termed uORFs and dORFs, respectively. Some studies suggest that uORFs may inhibit CDS translation, while dORFs may promote it, although these regulatory mechanisms are not fully clarified.

Recent MS results suggest that these sORFs may encode regulatory micropeptides [135]. The McKusick–Kaufman syndrome (MKKS) gene produces 2 transcripts: a long transcript containing both uORFs and MKKS, and a short transcript containing only uORFs produced via an alternative polyadenylation site in the 5′UTR. Researchers detected 2 uORF-encoded peptides, uMKKS1 and uMKKS2, in mitochondrial membrane fractions of HeLa cells. Furthermore, uMKKS1 and uMKKS2 were translationally translocated to the mitochondrial membrane to function [136]. In a recent study, uMKKS was also identified and confirmed to localize to mitochondria. Four other mitochondria-localized uORF-encoded micropeptides were simultaneously identified: uSLC35A4, iPGRMC1, uCGGBP1, and ouTFAM. Previous studies found that myeloid zinc finger 1 (MZF1) mRNA 5′UTR encodes MZF1-uPEP, which regulates glycolysis in NB [137]. Moreover, dORFs were found to encode micropeptides dCENPO and dREEP6, which localize to the cytoplasm and nucleus to perform various cellular functions [138]. In contrast, dORFs have been poorly studied. Similar to uORFs, dORFs often use non-AUG start codons, and their encoded micropeptides usually lack cross-species conservation [96,139].

Overall, the specific mechanisms of uORF and dORF translation remain unclear, and the precise functions of dORF-encoded micropeptides require further discovery.

## Biological Functions of NcRNA-Encoded Micropeptides in Cancer

Cancer is widely acknowledged for its high morbidity and mortality. The forecast predicts that by 2050, there will be 35 million new cases of cancer globally [140]. Published studies show that ncRNA-encoded micropeptides play key roles in the development of diverse cancers [14,141]. In terms of tumor metabolism, micropeptides modulate metabolic pathways and affect cancer cell proliferation and energy metabolism [14,111]. In tumor immunity, micropeptides regulate immune cell activity by enhancing or inhibiting immune response; for example, they affect T cell activity and immune checkpoint expression in the tumor microenvironment, influencing antitumor immunity (Table 5).

**Table 5.** Function of ncRNA-encoded micropeptides in cancer

Micropeptides	NcRNAs	Cancer	Functions	Ref.
circMRCKα-227aa	circMRCKα	HCC	Enhance glycolysis by inhibiting ubiquitination and degradation of HIF-1α	[147]
circPETH-147aa	circPETH	HCC	Promote glycolysis by enhancing PKM2 and ALODA interaction	[14]
ASAP	LINC00467	CRC	Increase ATP synthase activity and mitochondrial oxygen consumption rate	[149]
HOXB-AS3	HOXB-AS3	CRC	Suppress glycolysis by competitive binding to hnRNP A1 disrupts the binding of hnRNP A1 to PKM exon 9	[112]
pep-AP	lnc-AP	CRC	Inhibit pentose phosphate pathway	[150]
circINSIG1-121	circINSIG1	CRC	Induce cholesterol biosynthesis by promoting ubiquitination and degradation of INSIG1	[151]
hSPAR	LINC0096	BC	Inhibit glutamine uptake by regulation mTOR pathway	[152]
127aa	circSpdyA	BC	Promote de novo fatty acid synthesis by directly binding to FASN	[154]
circFAM53B-219	circFAM53B	BC	Enhance the infiltration of tumor antigen-specific cytotoxic T cells	[158]
H19-IRP	lnc-H19	GBM	Promote MDSCs, TAM infiltration, and T cell depletion	[160]
AMPK1-360aa	circAMPK	PC	Promote autophagy by inhibiting AMPK1 ubiquitination and degradation	[173]
N1DARP	LINC00261	PC	Suppress Notch1 signaling and chemoresistance by promoting Notch1 ubiquitination and degradation	[174]
RASON	LINC00673	PC	Promote tumorigenesis through interaction with KRAS^G12D/V^	[175]
GSPT1-238aa	circGSPT1	GC	Suppress cell invasion and migration	[176]
circDDX17-63aa	circDDX17	GC	Inhibit cell proliferation and migration	[177]
KEAP1-259aa	circKEAP1	OS	Inhibit cell stemness and metastasis by promoting vimentin proteasome degradation and activating antitumor immunity	[178]
LINC00665_18aa	LINC00665	OS	Suppress tumor growth by disrupting CREB1–RPS6KA3 interaction	[179]
CIP2A-BP	LINC00665	TNBC	Inhibit cell invasion and migration by suppressing PI3K/AKT/NFκB pathway	[180]
MAGI2-AS3-ORF5	MAGI2-AS3	BC	Inhibit cell proliferation and migration by suppressing MMP9	[181]
ASRPS	LINC00908	TNBC	Suppress angiogenesis by decreasing STAT3 phosphorylation levels	[182]

HIF-1α, hypoxia-inducible factor-1α; PKM2, pyruvate kinase M2; ALDOA, aldolase A; hnRNP A1, heterogeneous nuclear ribonucleoprotein A1; INSIG1, insulin-inducible gene 1; FASN, fatty acid synthase; MDSCs, myeloid-derived suppressor cells; TAMs, tumor-associated macrophages; AMPK1, AMP-activated protein kinase; Notch1, Notch receptor 1; CREB1, cAMP response element binding protein 1; RPS6KA3, ribosomal protein S6 kinase A3; MMP9, matrix metallopeptidase 9; STAT3, signal transducer and activator of transcription 3; HCC, hepatocellular carcinoma; CRC, colorectal cancer; BC, breast cancer; GBM, glioblastoma; PC, pancreatic cancer; GC, gastric cancer; OS, osteosarcoma; TNBC, triple-negative breast cancer

### NcRNA-encoded micropeptides regulate tumor metabolism

**Fig. 2.**
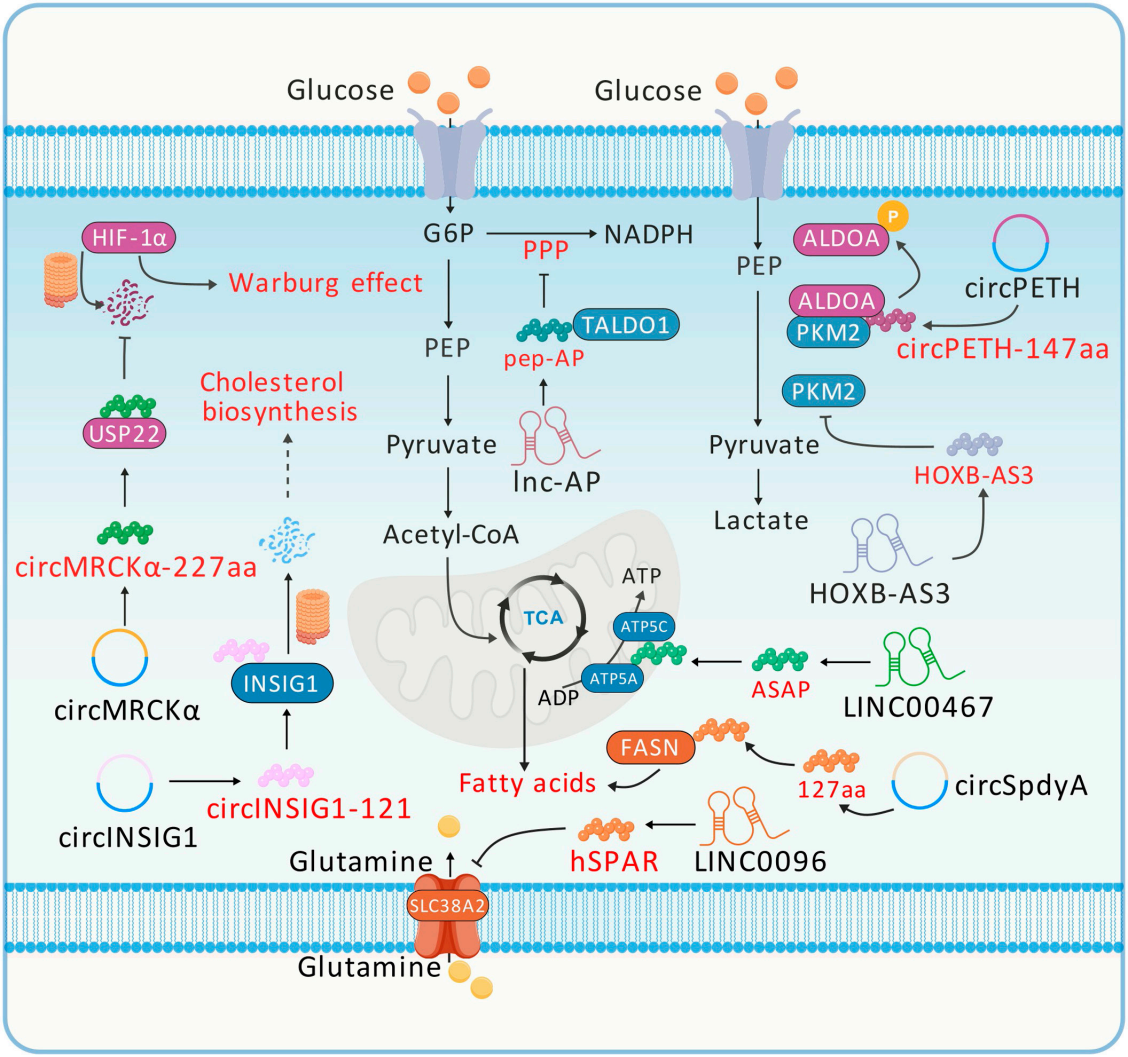
Regulation of tumor metabolism by ncRNA-encoded micropeptides. In HCC, circRNA-encoded micropeptides enhance glycolysis. In CRC, lncRNA-encoded micropeptides suppress glycolysis and pentose phosphate pathway and induce ATP synthase activity and cholesterol biosynthesis. In BC, ncRNA-encoded micropeptides inhibit glutamine uptake and promote de novo fatty acid synthesis.

A distinctive feature of cancer cells is altered metabolic patterns, enabling rapid growth and survival under unfavorable conditions [142]. The Warburg effect promotes glucose uptake and lactate production [143]. Oncogene activation and tumor suppressor gene mutations are key drivers. For example, MYC and RAS activation can elevate glycolytic enzyme expression [144]. Meanwhile, mutations in tumor suppressor genes such as the tumor protein P53 (TP53) and the phosphatase and tensin homolog (PTEN) further enhance glycolysis [145,146] (Fig. 2).

#### Hepatocellular carcinoma

Yu et al. [147] recently found that circMRCKα was up-regulated in tumor-associated macrophage (TAM)-treated HCC cells. Further evidence suggests that the circMRCKα-encoded micropeptide circMRCKα-227aa binds and stabilizes the deubiquitylating enzyme USP22, preventing hypoxia-inducible factor-1α (HIF-1α) degradation via the ubiquitin–proteasome pathway, enhancing glycolysis and HCC progression. Similarly, circPETH is highly expressed in HCC tissues, and its up-regulation correlates with tumor formation. It was revealed that the m^6^A modification of circPETH IRES drives translation of circPETH-147aa, which promotes the interaction of pyruvate kinase M2 (PKM2) and fructose bisphosphate aldolase A (ALDOA), enhancing aerobic glycolysis in HCC cells [14].

#### Colorectal cancer

CRC ranks second among causes of cancer death [148]. A previous study showed that ASAP, a 94-aa length micropeptide encoded by lncRNA LINC00467, plays a vital role in mitochondrial metabolism by increasing ATP synthase activity and mitochondrial oxygen consumption rate in CRC [149]. Moreover, lncRNA HOXB-AS3 is overexpressed in cancer cells and encodes a 53-aa micropeptide. Low expression of micropeptide HOXB-AS3 is associated with poor prognosis in CRC patients, which is explained by the fact that HOXB-AS3 micropeptide deficiency promotes aerobic glycolysis and CRC cell growth [112]. In another study, researchers found that lnc-AP encodes the 34-aa micropeptide pep-AP. Functionally, pep-AP inhibits the pentose phosphate pathway by interacting with specific proteins, reducing NADPH [reduced form of nicotinamide adenine dinucleotide phosphate (NADP^+^)]/NADP^+^ and glutathione (GSH) levels. This leads to increased reactive oxygen species (ROS) and apoptosis, enhancing CRC cell drug sensitivity [150]. Furthermore, Xiong et al. [151] found that circINSIG1 was up-regulated under hypoxic conditions in CRC cell lines and CRC tissue, and that its encoded novel protein circINSIG1-121 interacts with insulin-inducible gene 1 (INSIG1) to promote ubiquitylated degradation for cholesterol biosynthesis.

#### Breast cancer

A novel study found that the micropeptide human small regulator of amino acid response polypeptide (hSPAR), encoded by LINC0096, plays a key role in regulating breast cancer (BC) glutamine metabolism. The micropeptide hSPAR inhibits glutamine uptake and promotes P27KIP1 translocation from the cytoplasm to lysosome. Lysosomally localized P27KIP1 inhibits BC progression by disrupting mTORC1 complex assembly, blocking the mTOR signaling [152]. Recently, Gao et al. [154] found that circSpdyA encodes a 127-aa micropeptide that promotes de novo fatty acid synthesis [153] by directly binding to fatty acid synthase (FASN), driving BC tumor progression.

In summary, these studies reveal the essential functions of ncRNA-encoded micropeptides in regulating tumor glucose metabolism, mitochondrial metabolism, amino acid metabolism, and lipid metabolism.

### NcRNA-encoded micropeptides regulate tumor immunity

**Fig. 3.**
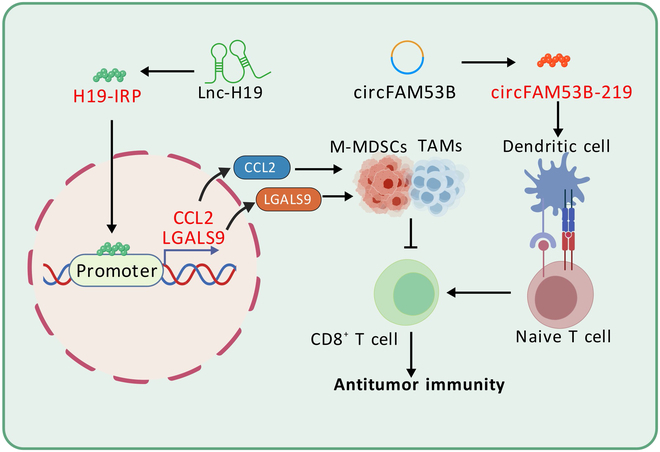
Regulation of tumor immunity by ncRNA-encoded micropeptides. NcRNA-encoded micropeptides enhance the infiltration of tumor antigen-specific cytotoxic T cells and promote MDSCs, TAM infiltration, and T cell depletion.

Immunotherapy has made significant progress in cancer treatment [155]. It recognizes and attacks tumor cells by activating or enhancing the patient’s immune system [156], offering advantages over traditional therapies. Immune checkpoint inhibitors (ICIs), such as anti-cytotoxic T lymphocyte-associated protein 4 (CTLA-4), anti-programmed death receptor 1 (PD-1), and anti-programmed death receptor ligand 1 (PD-L1), have been successfully applied clinically for various tumors [157]. By blocking immunosuppressive signaling, ICIs release the tumor’s suppression of the immune system, allowing T cells to regain antitumor function, resulting in durable remission (Fig. 3).

#### Breast cancer

A recent study showed that the 219-aa peptide circFAM53B-219, encoded by circRNA, activates and directs unstimulated T cells to promote immune cell attack on tumors [158]. CircRNA vaccines exhibit greater stability than mRNA vaccines due to their closed-loop structure and longer half-life [159]. They effectively stimulate immune responses against different diseases. For example, a vaccine using the peptide circFAM53B-219 encoded by circFAM53B can enhance the infiltration of tumor antigen-specific cytotoxic T cells, thereby achieving effective tumor control [158].

#### Glioblastoma

In malignant gliomas (GBM), lnc-H19 encodes the 256-aa peptide H19-IRP, which promotes myeloid-derived suppressor cells (MDSCs) and TAM infiltration, as well as T cell depletion, essential for forming an immunosuppressive GBM microenvironment. Furthermore, researchers cyclized H19 to develop the vaccine circH19-vac, which inhibits GBM progression in vivo by stimulating antitumor T cell responses through H19-IRP expression [160].

These findings highlight that peptides encoded by ncRNA can drive effective antitumor immunity. Targeting this for tumor-specific vaccine development is a promising immunotherapeutic strategy.

### NcRNA-encoded micropeptides regulate tumor invasion and migration

**Fig. 4.**
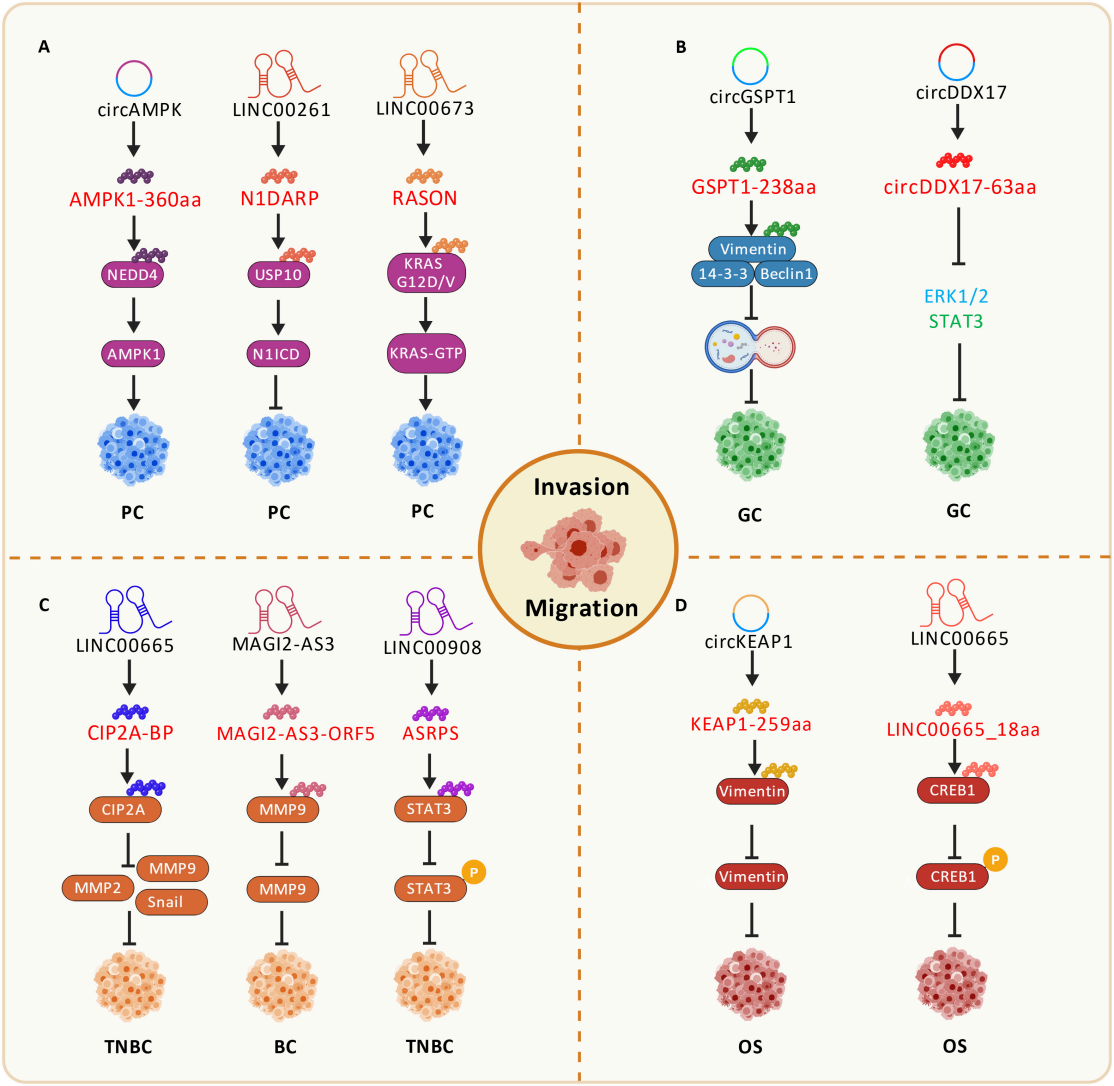
Regulation of tumor invasion and migration by ncRNA-encoded micropeptides. (A) In PC, micropeptide AMPK1-360aa and RASON drive tumorigenesis. However, the micropeptide N1DARP suppresses tumor development. (B) In GC, circRNA-encoded micropeptides inhibit cell invasion and migration. (C) In BC/TNBC, lncRNA-encoded micropeptides inhibit cell invasion and migration. (D) In OS, ncRNA-encoded micropeptides suppress tumor growth.

In addition to tumor immunity and metabolism, ncRNA-encoded micropeptides play essential roles in tumorigenesis and progression, particularly invasion and migration [161]. Invasion refers to tumor cells detaching from their origin and invading surrounding normal tissues. Cancer cells use various mechanisms to destroy surrounding tissue structure, enabling spread and metastasis [162]. Most cancers originate in epithelial tissue, and tumor cells remodel intercellular tight junctions and cell–matrix adhesion to gain migratory ability and invade neighboring tissues [163,164]. Tumor cells mainly migrate and spread through blood or lymphatic systems [165]. After detaching, cancer cells increase migratory capacity, damaging the extracellular matrix (ECM) [166] and basement membranes, forming invasive growths in normal tissues. Surviving cells move from vessel walls to grow in suitable tissues, forming metastatic foci that promote proliferation and trigger new metastases [167] (Fig. 4).

#### Pancreatic cancer

Pancreatic cancer (PC) is highly aggressive and lethal, with pancreatic ductal adenocarcinoma having a 5-year survival rate of only 13% [168–170]. Recent studies show that CD105-positive (CD105^+^) tumor-associated fibroblasts (CAFs) regulate AMPK1 stability and autophagy [171,172] via exosomally secreted circAMPK encoding AMPK1-360aa. This novel protein competitively binds the E3 ubiquitin–protein ligase NEDD4, protecting AMPK1 from ubiquitination and degradation, thereby promoting PC development [173]. Moreover, Zhai et al. [174] found that N1DARP, a 41-aa micropeptide encoded by LINC00261, competitively impedes USP10-mediated deubiquitination of the Notch1 intracellular structural domain (N1ICD), stabilizing N1ICD to inhibit tumor development. In another study, Cheng et al. [175] found by Ribo-seq that LINC00673 had the most significant translational differences in PC cell lines, encoding the novel protein RASON. Intriguingly, RASON directly binds KRAS^G12D/V^, maintaining it in a highly active state, thereby driving tumorigenesis.

#### Gastric cancer

Increasing evidence suggests that ncRNA-encoded micropeptides affect cancer cell invasion and migration through multiple mechanisms. For example, GSPT1-238aa, encoded by circGSPT1, substantially inhibited GC cell invasive and migratory ability. GSPT1-238aa regulates protein function and activity by interacting with specific proteins associated with tumor invasion and migration [176]. Moreover, Liu et al. [177] found that circDDX17 was down-regulated in GC and encodes circDDX17-63aa, which inhibits GC cell proliferation and migration. The exact mechanism by which the micropeptide circDDX17-63aa affects GC progression has not been reported. Overall, ncRNA-encoded micropeptides can directly influence the occurrence and development of tumor cells by regulating key proteins involved in invasion and migration.

#### Osteosarcoma

NcRNA-encoded micropeptides also play vital roles in cytoskeleton reorganization and dynamics, maintaining cellular morphology and motility. For example, Zhang et al. [178] found that KEAP1-259aa, encoded by circKEAP1, inhibits osteosarcoma (OS) cell invasion and migration by promoting vimentin degradation by binding to the E3 ubiquitin ligase ARIH1. Additionally, lncRNA LINC00665 encodes an 18-aa micropeptide, LINC00665_18aa, which inhibits human OS cell viability, proliferation, and migration. It hinders cAMP response element binding protein 1 (CREB1) binding to ribosomal protein S6 kinase A3 (RPS6KA3), thereby inhibiting tumor growth [179].

#### Breast cancer

Guo et al. [180] identified CIP2A-BP, a micropeptide encoded by LINC00665 in triple-negative breast cancer (TNBC) cells. CIP2A-BP expression was substantially down-regulated in TNBC patients. CIP2A-BP inhibited TNBC invasion and migration by suppressing the phosphatidylinositol 3-kinase (PI3K)/AKT/nuclear factor κB (NFκB) signaling pathway and exerted antitumor effects in mouse models, suggesting it is a promising therapeutic target and prognostic marker. Another study shows that MAGI2-AS3-ORF5, encoded by lncRNA MAGI2-AS3, inhibits BC progression, potentially by inhibiting the ECM degradation-related protein MMP9 [181]. Similarly, Wang et al. [182] identified ASRPS, a 60-aa micropeptide encoded by LINC00908 in TNBC cells. ASRPS inhibits BC cell invasion and migration by directly interacting with signal transducer and activator of transcription 3 (STAT3), reducing its phosphorylation level. Therefore, ASRPS can serve as a specific therapeutic target for TNBC.

In summary, these findings suggest that ncRNA-encoded micropeptides critically regulate tumor invasion and migration by affecting cytoskeleton stability, cellular morphology, and motility. In the aspect of tumor therapy, the discovery of these micropeptides provides a basis for developing novel approaches targeting them.

## Biological Functions of NcRNA-Encoded Micropeptides in Other Diseases

**Fig. 5.**
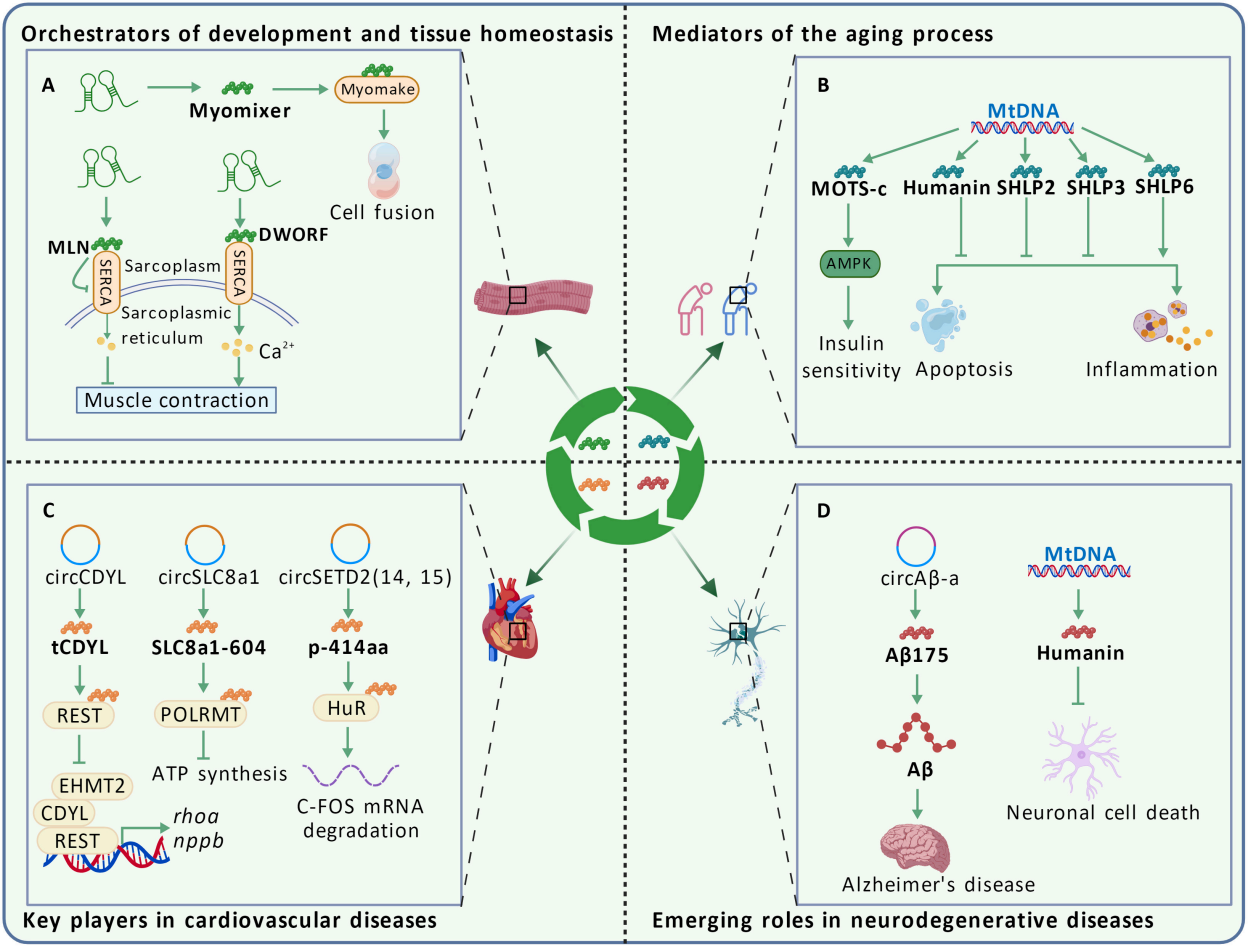
Function of micropeptides encoded by ncRNA in other diseases. (A) The micropeptide Myomixer interacts with the fusogenic membrane protein Myomake to synergistically induce cell fusion. The micropeptide DWORF promotes muscle contraction by interacting with SERCA, while micropeptide MLN suppresses muscle contraction via interaction with SERCA. (B) Mitochondrial rRNA-encoded micropeptide MOTS-c increases insulin sensitivity by modulating AMPK pathway. Micropeptide Humanin, SHLP2, and SHLP3 inhibit cell apoptosis or inflammation, while micropeptide SHLP6 promotes cell apoptosis or inflammation. (C) Micropeptides encoded by ncRNAs regulate gene transcription, ATP synthesis, and mRNA degradation in cardiovascular disease. (D) Emerging role for micropeptide Aβ175 and Humanin in neurodegenerative diseases.

In CVDs, ncRNA-encoded micropeptides regulate gene transcription or signaling pathways by interacting with specific proteins, thereby enhancing or inhibiting the onset and progression of CVDs. For example, certain encoded peptides play key roles in cardiac hypertrophy, atrial fibrillation (AF), atherosclerosis (AS) [110], and vascular remodeling [183]. In neurodegenerative diseases, a circAβ-encoded micropeptide Aβ175 has been identified in AD [184]. Additionally, ncRNA-encoded micropeptides have also demonstrated unique biological functions in other diseases and may become new targets for disease diagnosis and treatment [185] (Fig. 5).

### Orchestrators of development and tissue homeostasis

NcRNA-encoded micropeptides are essential for building and maintaining normal tissue function [186,187] (Fig. 5A). For example, Myomixer is critical for fusion and skeletal muscle formation during embryogenesis. The micropeptide Myomixer localizes to the cell membrane, promotes myogenic cell fusion, and interacts with the fusogenic membrane protein Myomake to synergistically induce fusion. In addition, the micropeptide Myomixer is up-regulated during embryonic development and muscle regeneration; its knockout in mice results in defective muscle development [98]. Similarly, the cardiac-specific micropeptide DWOR localizes to the sarcoplasmic reticulum (SR) membrane of cardiomyocytes and binds to sarco-endoplasmic reticulum Ca^2+^ adenosine triphosphatase (SERCA), promoting its activity. The researchers found that hearts overexpressing DWORF showed increased SERCA activity, while DWORF deficiency led to a decreased affinity of SERCA for calcium. In skeletal muscle, DWORF deficiency affects muscle recovery from tonic contraction, indicating its importance for recovery after prolonged contraction and calcium release [109]. In another study, the micropeptide myoregulin (MLN) was specifically expressed in skeletal muscle. MLN directly interacts with SERCA, impeding calcium ion entry into the SR. *MLN* knockout in mice enhances skeletal muscle calcium handling and improves exercise performance [186]. Collectively, these findings emphasize that ncRNA-encoded micropeptides maintain developmental and tissue homeostasis by regulating membrane protein activity.

### Mediators of the aging process

Age-related diseases such as type 2 diabetes (T2D), coronary artery endothelial dysfunction, and AD are closely related to ncRNA-encoded micropeptides [8] (Fig. 5B). They can serve as key molecules regulating aging rate and aging-related phenotypes (e.g., cellular senescence [188], mitochondrial dysfunction, and inflammation). The role of MDPs in aging is most extensively studied. MOTS-c, originally considered an exercise mimetic peptide, reduces obesity and insulin resistance by modulating the folate–purine–AMPK pathway [189]. Besides, exercise-induced MOTS-c in humans regulates nuclear genes related to metabolism and proteostasis, skeletal muscle metabolism, and myocyte adaptation to metabolic stress. Intriguingly, MOTS-c treatment improved physical performance and healthy lifespan in mice [190]. Humanin, originally identified in the occipital lobe of AD patients’ brains, protects neurons from amyloid-β (Aβ) toxicity [191]. Meanwhile, other studies have demonstrated that the humanin acts as a cytoprotective factor, inhibiting apoptosis by binding to the pro-apoptotic molecules IGFBP3 and BAX [192,193]. Additionally, humanin prevents synaptic loss in hippocampal neurons and reduces the inflammatory response [194]. SHLPs are MDPs sharing biological properties with humanin. For example, SHLP2 inhibits islet amyloid polypeptide (IAPP) misfolding and exerts cytoprotective effects [195]. Circulating levels of SHLP2 decrease significantly with age, supporting its potential role in aging and age-related diseases. SHLP3 improves mitochondrial metabolism, reduces ROS production, and regulates AKT and AMPKα for cytoprotection [196,197]. SHLP3 also regulates the inflammatory response by up-regulating inflammatory markers interleukin-6 (IL-6) and monocyte chemotactic protein-1 (MCP-1). In contrast to SHLP2 and SHLP3, SHLP6 increases apoptosis [197].

### Key players in CVDs

CVD poses a major global health burden and is the leading cause of death worldwide. Pathological cardiac hypertrophy primarily results from cardiomyocyte death, chronic hypertension, or genetic defects, accompanied by enlarged cardiomyocytes, asymmetric ventricular thickening, cardiac fibrosis, and hypoplasia. It can lead to heart failure and death [198]. In primary rat cardiomyocytes, circCDYL was associated with cardiac hypertrophy and induced by Ang II. Li et al. [199] reported that circCDYL is translated into the 100-aa truncated chromodomain Y-like peptide (tCDYL-100) in an m^6^A-dependent manner. Their study identified a novel signaling pathway for pathological cardiac hypertrophy: m^6^A-circCDYL-tCDYL-100-REST (RE1-silencing transcription factor)/CDYL-RhoA (Ras homolog family member A)/BNP (brain natriuretic peptide). The micropeptide tCDYL-100 competes with full-length CDYL for binding cytoplasmic REST proteins. This competition prevents REST from forming REST-CDYL-EHMT2 (euchromatic histone-lysine N-methyltransferase 2) complex, activating rhoa and brain natriuretic peptide (nppb) and other pro-hypertrophic gene transcription. This finding provides a potential new target for the treatment of pathological cardiac hypertrophy. In a newly published study, Li et al. [200] found that human circSLC8a1 encodes SLC8a1-604, detected only in human heart tissue. SLC8a1-604 binds to mitochondrial RNA polymerase POLRMT, inhibiting mitochondrial gene transcription, decreasing ATP synthesis, and ultimately reducing cardiac function. AF is one of the most important types of CVD [201]. Du et al. [202] found that circNAB1 is down-regulated in AF specimens from patients and encodes NAB1-356. NAB1-356 interacts with EGR1 to reduce atrial fibrosis and inflammation. Additionally, NAB1-356 regulates the transcription of transcription factors Runx1 and Gadd45b, affecting cytokine expression and fibrosis.

AS is another CVD that is characterized by lipid accumulation, inflammatory response, and fibrosis within the arterial wall, leading to thickening, hardening, and decreased elasticity of the vessel wall. Phenotypic switching of VSMCs is the underlying cause of vascular remodeling diseases such as AS and hypertension [203]. VSMCs play a core role in the onset and development of AS, and their functional abnormalities directly drive plaque formation and disease progression [204]. Yu et al. [110] found that lncPSR expression was up-regulated in VSMCs after Ang II treatment and confirmed that lncPSR encodes Arteridin, a novel 117-aa protein. Arteridin directly interacts with the transcription factor YBX1 to regulate downstream gene expression, thereby inducing VSMC phenotypic shift. Accumulating evidence suggests that Arteridin is a potential therapeutic target for vascular remodeling associated with VSMC phenotypic switching. A recent study showed that overexpression of circSETD2 (14, 15), which encodes a novel protein p-414aa that binds to human antigen R (HuR) and promotes C-Fos proto-oncogene (C-FOS) mRNA degradation, inhibits VSMC proliferation by suppressing phenotypic transition [183] (Fig. 5C).

### Emerging roles in neurodegenerative diseases

Neurodegenerative diseases encompass disorders caused by neuronal dysfunction [205]. AD is one of the most common neurodegenerative disorders characterized by an abnormal accumulation of extracellular Aβ plaques and intracellular neurofibrillary tangles in the brain [206], which manifests itself as cognitive dysfunction in older adults, such as memory loss, abnormal thinking, and mental retardation [207]. Mo and colleagues [184,208] demonstrated that circAβ-a encodes the micropeptide Aβ175 in cultured cells and human brain. CircAβ-a is derived from the Aβ coding region of the Aβ precursor protein (APP) gene and expressed in AD patients and healthy brains. Expression of the micropeptide Aβ175 is promoted in brain tissue of AD patients and may be associated with its pathogenesis by an unknown mechanism. The micropeptide Aβ175 is further processed into Aβ peptides, markers of AD pathogenesis. The discovery of the micropeptide Aβ175 provides a more comprehensive understanding of Aβ protein synthesis. Thus, circAβ-a and Aβ175 may be potential new targets for AD therapy. Additionally, Hashimoto et al. [209] found that HN, encoded by mitochondrial 16S rRNA, eliminated neuronal cell death induced by multiple familial AD genes and Aβ amyloid, suggesting HN as a new target for neuroprotective AD therapies. In neurodegenerative diseases, most predicted ncRNA-encoded micropeptides lack experimental validation, requiring more sensitive MS techniques and antibody tools (Fig. 5D).

Taken together, these findings emphasize that ncRNA-encoded micropeptides play key roles in various pathological processes, including neurodegenerative and cardiovascular. They not only are involved in pathogenesis but also represent potential therapeutic targets.

## Regulatory Mechanisms of NcRNA-Encoded Micropeptides in Disease

**Fig. 6.**
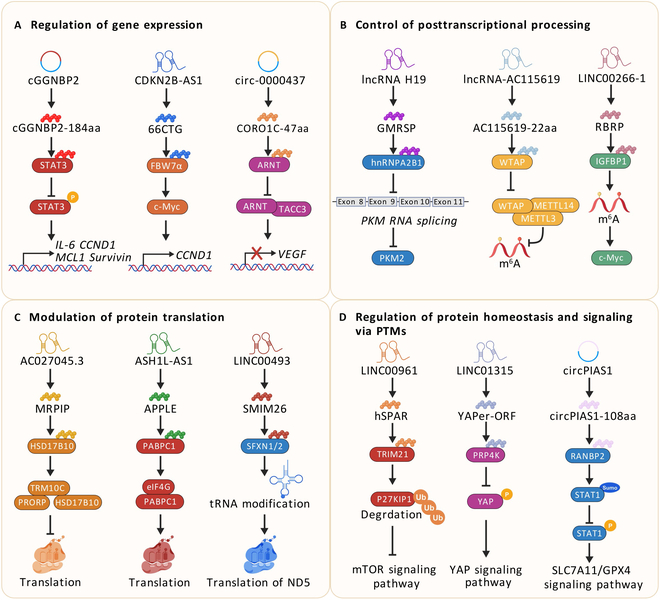
Regulatory mechanisms of micropeptides encoded by ncRNA in diseases. (A) NcRNA-encoded micropeptides bind to transcription factors or their partners to regulate gene expression. (B) NcRNA-encoded micropeptides control posttranscriptional processing, such as RNA splicing, RNA modification, and stability. (C) NcRNA-encoded micropeptides regulate protein translation by influencing translation-related complexes. (D) NcRNA-encoded micropeptides regulate protein homeostasis and signaling via PTMs, such as ubiquitination, phosphorylation, SUMOylation, and acetylation.

The disease-associated translatable ncRNAs encode micropeptides that influence disease development through various mechanisms regulating cellular biological processes [210] (Table 6), signaling pathways, protein function, and gene expression (Fig. 6).

**Table 6.** Molecular mechanisms of ncRNA-encoded peptides in disease

Micropeptides	NcRNAs	Diseases	Mechanisms	Ref.
cGGNBP2-184aa	cGGNBP2	ICC	Activate JAK–STAT pathway by interaction with STAT3	[211]
HDSP	HOXA10-HOXA9	GC	Activate MECOM–SPINK1–EGFR signaling axis by binding MECOM	[212]
66CTG	CDKN2B-AS1	TNBC	Promote cyclin D1 transcription by inhibition c-Myc ubiquitination and degradation	[213]
PINT87aa	LINC-PINT	GBM	Suppress PHB2 gene transcription by binding to FOXM1	[214]
CORO1C-47aa	circ-0000437	EC	Reduce VEGF expression by binding to ARNT	[215]
GMRSP	H19	AD	Inhibit PKM alternative splicing by interacting with hnRNPA2B1	[216]
PRDM16-DT	LINC00982	CRC	Promote L-CHEK2 splicing by competitively binding hnRNPA2B1	[217]
SRSP	LOC90024	CRC	Induce oncogenic L-Sp4 protein splicing via interaction with SRSF3	[218]
TPM3P9	TPM3P9	ccRCC	Drive TCF7L2-L splicing and tumorigenesis by binding to RBM4	[113]
AC115619-22aa	lncRNA-AC115619	HCC	Disrupt METTL3–METTL14 complex assembly via interaction with WTAP	[220]
RBRP	LINC00266-1	CRC	Stabilize c-Myc mRNA by interacting with m^6^A reader IGF2BP1	[244]
HCP5-132aa	HCP5	GC	Promote SLC7A11/G6PD mRNA m^5^C modification by binding to YBX1 and ELAVL1	[223]
MRPIP	AC027045.3	HCC	Suppress mitochondrial RNA translation by disrupting mtRNase P complex assembly	[77]
APPLE	ASH1L-AS1	AML	Promote mRNA circularization and eIF4F initiation complex assembly by enhancing PABPC1–eIF4G interaction	[226]
SMIM26	LINC00493	AML	Promote ND5 protein translation by binding to mitochondrial serine transporter SFXN1/2 and mitochondrial ribosome	[99]
circPCSK6-167aa	circPCSK6	ICC	Inhibit NFκB pathway through regulation of IκB stability and ubiquitination	[229]
circZKSaa	circZKSCAN1	HCC	Inhibit AKT pathway by enhancing mTOR ubiquitination	[230]
YAPer-ORF	LINC01315	UM	Hijack YAP signaling via competitive binding to phosphokinase PRP4K	[231]
YAP-220aa	circ-YAP	CRC	Activate metastasis-promoting genes by interaction with phosphokinase LATS1	[11]
AKT3-174aa	circ-AKT3	GBM	Inhibit PI3K–AKT pathway by competitive binding to PDK1	[233]
PDHK1-241aa	circPDHK1	ccRCC	Activate AKT–mTOR pathway by binding to phosphatase PPP1CA	[234]
ZNF609-250aa	circ-ZNF609	AKI	Activate AKT–mTOR pathway through up-regulation AKT3	[122]
C-E-Cad	circ-E-Cadherin	GBM	Activate STAT3 pathway by binding to EGFR	[235]
circPIAS1-108aa	circPIAS1	MM	Enhance STAT1 SUMOylation by binding to SUMO E3 ligase RANBP2	[237]
pTINCR	TINCR	cSCC	Enhance CDC42 SUMOylation via interaction with CDC42	[238]
SLC9A6-126aa	circ-SLC9A6	NAFLD	Promote CD36 expression by recruiting acetyltransferase MOF to enhance H4K16ac	[239]
ACLY-BP	LINC00887	ccRCC	Stabilize ACLY by maintaining acetylation and preventing ubiquitylation and degradation	[240]

ICC, intrahepatic cholangiocarcinoma; AD, aortic dissection; L-CHEK2, long CHEK2; L-Sp4, long Sp4 isoform; TCF7L2-L, long splice variant TCF7L2; mtRNase P, mitochondrial ribonuclease P; AML, acute myeloid leukemia; AKI, acute kidney injury; UM, uveal melanoma; MM, malignant melanoma; cSCC, cutaneous squamous cell carcinoma; NAFLD, nonalcoholic fatty liver disease

### Regulation of gene expression

A growing number of studies show that ncRNA-encoded micropeptides can influence gene transcription by binding to transcription factors or their partners (Fig. 6A). For example, circRNA GGNBP2 (termed as cGGNBP2) encodes cGGNBP2-184aa, which directly interacts with STAT3 to promote Tyr^705^ phosphorylation, facilitating downstream gene transcription [211]. Additionally, the micropeptide HDSP encoded by lncRNA HOXA10-HOXA9 binds transcription factor MECOM, preventing its ubiquitination and degradation by E3 ubiquitin ligase TRIM25, thereby promoting serine protease inhibitor Kazal type 1 (SPINK1) transcription [212]. Similarly, micropeptide 66CTG encoded by lncRNA CDKN2B-AS1 competitively binds to the E3 ubiquitin ligase FBW7α subunit, stabilizing the transcription factor c-Myc to promote cyclin D1 transcription [213]. Conversely, the micropeptide PINT87aa encoded by LINC-PINT binds to the transcription factor FOXM1, thereby blocking prohibitin 2 (PHB2) gene transcription [214]. Similarly, the micropeptide CORO1C-47aa encoded by hsa-circ-0000437 inhibits vascular endothelial growth factor (VEGF) expression by competing with the transcription factor TACC3 for binding to the aryl hydrocarbon receptor nuclear translocator (ARNT) [215].

### Control of posttranscriptional processing

#### RNA splicing

NcRNA-encoded micropeptides precisely regulate alternative splicing by binding to splicing factors in various diseases (Fig. 6B). For example, in aortic coarctation disease, a recent study demonstrated that the small protein glucose metabolism regulatory protein (GMRSP), encoded by lncRNA H19, binds to the glycine-rich structural domain of hnRNPA2B1, inhibiting hnRNPA2B1-mediated alternative splicing of pyruvate kinase M (PKM) pre-mRNA, reducing PKM2 production and glycolysis [216]. Similarly, PRDM16-DT encoded by LINC00982 directly interacts with hnRNPA2B1 and competitively reduces the binding of the hnRNPA2B1 phase to exon 9 of checkpoint kinase 2 (CHEK2), leading to long CHEK2 (L-CHEK2) formation in CRC [217]. In addition, the small protein SRSP, encoded by the long noncoding RNA LOC90024, interacts with the splicing factor SRSF3. This interaction promotes the binding of SRSF3 to the third exon of the transcription factor Sp4, which increases expression of the oncogenic long isoform (L-Sp4) and suppresses the non-oncogenic short isoform (S-Sp4) [218]. Recent studies in ccRCC found that lncRNA tropomyosin 3 pseudogene 9 encodes a novel protein, TPM3P9, which interacts with splicing factor RNA-binding motif protein 4 (RBM4), inhibiting RBM4-mediated exon skipping of transcription factor 7-like 2 (TCF7L2), resulting in increased long splice variant TCF7L2-L expression [113].

#### RNA modification and stability

RNA modifications accompany the entire transcription process, affecting various biological processes and cellular phenotypes [219]. NcRNA-encoded micropeptides critically regulate RNA modification by binding RNA modification writers or readers (Fig. 6B). For example, AC115619-22aa, encoded by lncRNA-AC115619, binds to m^6^A writer WT1-associated protein (WTAP). This binding disrupts WTAP-methyltransferase-like protein 3 (METTL3)–methyltransferase-like protein 14 (METTL14) complex formation, reducing overall m^6^A modification in tumor cells, inhibiting HCC cell growth [220]. In another study, Zhu et al. [221] found that the peptide RBRP encoded by lncRNA LINC00266-1 interacts with the m^6^A reader IGF2BP1, enhancing IGF2BP1 recognition m^6^A-modified RNAs, thus stabilizing and promoting c-Myc mRNA expression and driving tumorigenesis. Ferroptosis is a key mechanism involved in disease progression [222]. In GC, the micropeptide HCP5-132aa encoded by lncRNA HCP5 acts as a scaffolding protein to enhance m^5^C reader Y-box binding protein 1 (YBX1) binding to ELAV-like RNA-binding protein 1 (ELAVL1), promoting YBX1-mediated solute carrier family 7 member 11 (SLC7A11)/glucose-6-phosphate dehydrogenase (G6PD) mRNA m^5^C modification. Increased SLC7A11 and G6PD mRNA stability suppresses ferroptosis, driving GC progression [223].

#### Modulation of protein translation

NcRNA-encoded micropeptides regulate translation by competitively binding specific proteins (Fig. 6C). Recently, researchers identified many sORF-encoded micropeptides in clinical HCC samples using ultrafiltration tandem MS. AC027045.3 is down-regulated and encodes a mitochondrial RNase P inhibitory peptide (MRPIP) in HCC. MRPIP interacts with 3-hydroxyacyl-coenzyme A dehydrogenase type-2 (HSD17B10) at the R25 residue, impeding mitochondrial ribonuclease P (mtRNase P) complex assembly. This disrupts HSD17B10 tetramerization and subsequent HSD17B10–TRMT10C subcomplex formation, blocking mitochondrial RNA translation [77]. In acute myeloid leukemia (AML) [224], Sun et al. found that the micropeptide APPLE, encoded by lncRNA ASH1L-AS1, binds polyadenylate-binding protein cytoplasmic 1 (PABPC1) and facilitates the interaction between PABPC1 and the eukaryotic translation initiation factor 4G (eIF4G) [225], which promotes mRNA cyclization and eIF4F initiation complex assembly. The validation performed on human primary cells also showed that APPLE affects the translation initiation, but not the transcription [226]. Recently, Nah et al. [99] found that mitochondrial micropeptide small integral membrane protein 26 (SMIM26), encoded by lncRNA LINC00493, binds to mitochondrial serine transporter SFXN1/2 and the mitochondrial ribosome, promoting the electron transport chain (ETC) [227] complex I subunit mt-ND5 protein translation. The understanding of protein translation regulation mechanisms by ncRNA-encoded micropeptides remains poor, and it is therefore crucial to expand the understanding of the mechanisms of protein translation regulation by ncRNA-encoded micropeptides.

### Regulation of protein homeostasis and signaling via PTMs

#### Ubiquitination and degradation

Ubiquitination is a crucial posttranslational modification (PTM) regulating protein stability and function. E3 ubiquitin ligases and deubiquitinases dynamically and reversibly regulate ubiquitination modifications [228]. A series of studies demonstrated that ncRNA-encoded micropeptides regulate downstream signaling by binding to E3 ubiquitin ligase to promote or impede substrate protein ubiquitination and degradation (Fig. 6D). For example, the micropeptide hSPAR encoded by LINC00961 competitively binds to the E3 ubiquitin–protein ligase TRIM21, inhibiting TRIM21-mediated ubiquitination modification of cell-cycle protein-dependent kinase inhibitor 1B (P27KIP1) proteins to stabilize their expression, and inhibiting mTOR signaling [152]. Additionally, circPCSK6 encodes circPCSK6-167aa, which competitively binds E3 ubiquitin ligase RBBP6, inhibiting ubiquitinated degradation of NFκB inhibitor α (IκBα) to maintain its stability and regulate downstream signaling [229]. Similarly, the novel peptide circZKSaa encoded by circZKSCAN1 interacts with the E3 ubiquitin–protein ligase F-box and the WD repeat structural domain-containing 7 (FBXW7), promoting ubiquitinated degradation of mTOR to inhibit downstream signaling [230].

#### Phosphorylation and signaling cascades

Phosphorylation modifications and signaling cascades are crucial for cellular signaling, regulating physiological processes by altering protein activities and interactions. Several critical signaling pathways often show aberrant activation during disease, such as the Hippo/YAP pathway, the PI3K/AKT/mTOR pathway, and the STAT3 pathway. Studies show that ncRNA-encoded micropeptides can promote or inhibit these pathways by binding to phosphatases or kinases (Fig. 6D). For example, the micropeptide YAPer-ORF encoded by LINC01315 competitively binds to the intranuclear phosphokinase PRP4K of YAP1, inhibiting the phosphorylation modification of the transcriptional co-activator YAP, thereby activating the YAP signaling pathway [231]. Recently, Zhao et al. [232] found that lncRNA ASH1L-AS1 encodes APPLE, which interacts with extracellular signal–regulated kinase 1/2 (ERK1/2) and phosphatase PP1/PP2A, preventing ERK1/2 dephosphorylation and thereby activating the mitogen-activated protein kinase (MAPK) pathway. Additionally, circ-YAP encodes the oncogenic protein YAP-220aa, which interacts with phosphokinase large tumor suppressor kinase 1 (LATS1) to inhibit YAP phosphorylation, negatively regulating the Hippo/YAP pathway [11]. Similarly, circ-AKT3 encodes AKT3-174aa, which directly competes for binding to pyruvate dehydrogenase kinase 1 (PDK1) to reduce phosphorylation of the AKT-Thr^308^ site, thus negatively regulating the PI3K/AKT signaling pathway [233]. Moreover, the micropeptide PDHK1-241aa encoded by circPDHK1 inhibits AKT dephosphorylation by interacting with the serine/threonine protein phosphatase PPP1CA, thereby activating the AKT/mTOR pathway [234]. In another study, ZNF609-250aa encoded by circ-ZNF609 promotes mTOR phosphorylation by up-regulating AKT3 expression, activating the AKT/mTOR pathway [122]. Furthermore, C-E-Cad, encoded by circ-E-Cadherin, binds tyrosine kinase epidermal growth factor receptor (EGFR) to enhance STAT3 phosphorylation, activating the STAT3 pathway [235].

#### Other modifications

In addition to ubiquitination and phosphorylation, ncRNA-encoded micropeptides regulate other modifications like SUMOylation and acetylation. SUMO proteins are ubiquitin-like proteins covalently modifying other proteins via SUMOylation to regulate cellular processes. SUMO E3 ligase, such as PIAS family proteins, TRIM family members, and RAN-binding protein 2 (RANBP2), promote SUMO binding to target proteins [236]. NcRNA-encoded micropeptides regulate protein homeostasis and signaling by binding to specific proteins or SUMO E3 ligases (Fig. 6D). For example, the micropeptide circPIAS1-108aa encoded by circPIAS1 binds to the SUMO E3 ligase RANBP2, enhancing SUMOylation of signal transducer and activator of transcription 1 (STAT1) to inhibit its phosphorylation and regulate downstream signaling [237]. Similarly, the micropeptide pTINCR encoded by TINCR interacts with cell division control protein 42-homolog (CDC42) to enhance CDC42 SUMOylation, promoting its activation, epithelial differentiation, and tumor growth inhibition [238]. Additionally, ncRNA-encoded micropeptides regulate protein homeostasis and signaling via acetylation. For example, the micropeptide SLC9A6-126aa encoded by circ-SLC9A6 recruits the acetyltransferase MOF to promote H4K16ac, thereby activating the expression of the target gene CD36 [239]. Besides, LINC00887 encodes the micropeptide ACLY-BP, which directly interacts with ATP citrate lyase (ACLY) to promote acetylation at sites K540, K546, and K554, thereby stabilizing ACLY to regulate fatty acid synthesis [240]. Lactylation modification is a recently discovered PTM that regulates protein stability and downstream signaling in various cancers [241]. The exploration of ncRNA-encoded micropeptides to influence protein homeostasis in vivo by regulating lactylation modifications would be a novel direction.

In summary, ncRNA-encoded micropeptides play key roles in disease processes. We should broaden our perspective beyond classic ORF-encoded proteins and emphasize exploring the functional significance of ncRNA-encoded micropeptides to comprehensively analyze gene roles in disease development.

## Conclusions and Future Perspectives

NcRNA-encoded micropeptides play crucial roles in disease. Previously, ncRNAs were believed noncoding and mere transcription byproducts. However, advancements in ribosome-related sequencing and MS have identified several ncRNA-encoded micropeptides. Concurrently, several databases have been established, providing direct evidence for discovering new ncRNA-encoded micropeptides, such as the HMPA database [65]. New database establishment will facilitate a comprehensive understanding of ncRNA-encoded micropeptides. These micropeptides are key players in diverse physiological and pathological processes and participate in disease development through multiple mechanisms. They bind specific proteins, influencing RNA modification, transcription, and splicing processes, thereby regulating gene expression. It has been demonstrated that the lncRNA-encoded micropeptide MRPIP binds to HSD17B10 to regulate mitochondrial RNA translation [77]. Besides, ncRNA-encoded micropeptides regulate protein translation and posttranslational modifications, participating in cellular metabolism, immune response, and signaling. For instance, some ncRNA-encoded micropeptides modulate tumor metabolism, immune response, and cell invasive migration in cancer, affecting tumorigenesis and progression. In CVDs, micropeptides influence pathological processes such as cardiac hypertrophy, AS, and vascular remodeling by regulating gene transcription or signaling pathways. Dysregulated protein translation alters protein expression, function, and cellular homeostasis, playing key roles in numerous human diseases [242]. The lncRNA-encoded micropeptide APPLE has been reported to regulate translation initiation, affecting malignancy development [226]. However, mining micropeptides encoded by other translatable ncRNAs holds tremendous research value for translation regulation. Notably, PTM of ncRNA-encoded micropeptides is a promising new direction for exploration. The discovery of phosphorylation, acetylation, and ubiquitination modifications of micropeptides can deepen our understanding of their stability and function in disease development. Subsequent studies need to explore the mechanisms of these micropeptide PTMs and their impact on cellular processes. Recent studies have found that micropeptides can also bind to RNA to regulate mRNA stability, which opens up new directions for understanding their biological functions [243]. Future in-depth exploration of interactions between ncRNA-encoded micropeptides and nucleic acids will help reveal more disease-related regulatory networks and provide new diagnostic and therapeutic targets.

In conclusion, ncRNA-encoded micropeptides are indispensable mediators of disease biology. Ongoing technological innovations will propel a deeper and more comprehensive deciphering of the functional landscape of these micropeptides. This not only aids in better understanding disease mechanisms but also provides directions for developing diagnostic markers and therapeutic targets. Future research will require concerted breakthroughs in technology, mechanistic insight, and translation to accelerate the field. Elucidating the functional repertoire of micropeptides is critical for deciphering the pathogenic mechanisms driving disease initiation and progression.
